# Tri-State Meeting

**Published:** 1898-12

**Authors:** 


### PROCEEDINGS.

### Tri-State Meeting.

[*Continued from September No., Page* 438.]

Meeting called to order Wednesday morning at 9 o'clock. Adjourned to hotel parlors from assembly room.

J. R. CALLAHAN: Gentlemen, with the permission of the President, I will state the meeting this evening will begin promptly at 8 o'clock. We will lock the door at a certain time, and the house will be kept quiet for the benefit of the people present. After the concert is over there will be an informal reception, in which there will be music and refreshments. This will take place in the hall to this room. The Indiana State Dental Association will meet after this afternoon's session.

DR. LOEFFLER, Saginaw, Mich.: I would like at this time to make a motion that we extend a vote of thanks to Dr. Barrett, who so kindly consented to come to this meeting and give us that excellent paper. Adopted.

Discussion on paper opened by DR. TAFT: Mr. Chairman, I have simply this suggestion to make. The time is passing rapidly and the program is being reached slowly, and I would suggest that we proceed to the next order of business; and for another reason, that Dr. Barrett's lecture so fully covered the ground. There may be points in which persons would differ, but a profitable discussion would occupy two or three hours. I suggest that we proceed with the order of business. Motion made and carried.

"ACTION OF HEAT UPON DENTINE."

Dr. George W. Cook, Chicago, Ills.: Ladies and Gentlemen:—The effects of heat on cell life, especially micro-organisms, have been studied by many investigators. All found that the bacilli were not killed by moderate heat. Pasteur also took up the experiments, but did not give the full process of his method used. To Koch and his assistants we owe our thanks. In all his experiments the anthrax bacillus was employed, because of its greater resistance. Koch, Loeffler and others found that at 42° the virulence of the bacillus perceptibly diminished. The lower the temperature the longer time it took, but it is more permanent in its effects. The variation of a fraction of a degree is of importance. Nine days at 43° C. the anthrax bacillus was rendered harmless; forty days at 42.6°. On this fact I began a series of investigations embracing three factors. 1. Effects of heat on bacteria. 2. Amount of moisture extracted from dentine. 3. Effect of heat on dentine. The first is of great importance; it has been shown a temperature only 6° above body temperature will render micro-organisms harmless. Ninety-two degrees C. in most cases will kill bacteria in pulp canals, *i. e.*, heat applied for about ten minutes by means of root-canal dryer. Experiments at various degrees of heat were tried, and it was found that a temperature of 130° C. of three applications of four and one-half minutes each were most effective.

Dr. Black considers free water is an essential element in tooth structure. It is hard to tell what he means by that statement. If water is an element in the structure of dentine, it can not be in the form of water of crystallization. The inorganic matter does not require water for crystallization. All living tissue contains more or less water, but it does not enter into the chemical constituents of the molecule but is simply held by colation—the power of a cold substance to imbibe a certain amount of water. It might be stated that the function of water is to keep the colloid material in an elastic condition. Water is not an element in tooth structure at all, but its presence is necessary for the normal elasticity and to the functions of tooth tissue.

As the greater part of a tooth is composed of dentine, we will consider its structure and chemistry. We have twenty-eight parts of animal matter and seventy-two parts inorganic. The animal constituents are converted into gelatine on boiling. Calcium, magnesium and sodium phosphates make up the inorganic, and since none of these salts require the presence of water for crystalline formation, none of the moisture found can be considered water of crystallization. Also as none of these inorganic salts are affected, we must attribute all effects of temperature, varying from 70° to 130° C., to its action on the organic substances, of which there are about 28 percent. The colloids hold the inorganic salts together. It is a well-known fact that all colloid substances take up a certain percent of water by colation, and elasticity is due to this property. A shrinkage follows if water is taken off, and the tooth becomes brittle and inelastic; this is the case with old dry teeth and is explained by the dryness. It is hard to make out any contraction in dentine, but it is a well proven fact that after drying a tooth the enamel is easily broken off. This can only be explained by the removal of the water, thus rendering the organic structure brittle and inelastic. The presence of water, then, you see, is of great importance.

By the use of 125° C. for ten minutes on tooth sections, $\frac{1}{100}$ of an inch of dentine and enamel, I was unable to corroborate Dr. Black, that such treatment caused contraction of dentine. I could not see such a separation of dentine from enamel—the dentine was more porous and enamel more brittle.

Dr. Black also used 200° F. for twelve hours and found contraction, while I could not detect a contraction. From these facts it is possible to determine the effects of temperature on bacteria in from ten to twenty minutes in tooth structure. I found that the application of a temperature of 130° C. in three applications of four and one-half minutes each was sufficient to kill bacteria. This treatment gradually reduced the weight of the tooth from .0065 to 1 percent, due to loss of water. This loss of water also aids in destruction of bacteria. The question of its resistance power on tooth structure must also be taken into consideration. The temperature has no effect on the inorganic salts in the teeth.

The results of my experiments as made on tooth sections all show that they are rendered more brittle by so high a temperature as 130° C. for ten minutes. Of course, there is a difference in the manner of application of heat and in form of material acted upon. In the second case we had a small mass and 1 percent of moisture driven off. The colloid substances are rendered hard and brittle. After removing all moisture possible by mechanical means it is well to take root-dryer heat from 90° to 130° C and apply for a very few minutes, but if temperature be raised to where you get the hissing sound, we get a drying out of the tooth which renders it more brittle and unsatisfactory.

DISCUSSION.

Discussion opened by Dr. Kahlo, of Indianapolis. Mr. President:—The essayist has given us a very interesting and entertaining paper. While I do not concur with all his views on this subject, I heartily thank him for the excellent paper. Most of his experiments were made on tissues out of the mouth; the conditions present in different mouths are vastly different. In reference to presence of water in tooth structure, I think water exists as free water; if such were not the case how could we explain the fact that after drying out the root canal but a short time elapses until it returns to its natural condition? Water probably influences the toughness and firmness of the tooth. For these reasons I see no objection why it should not be considered as a part of the structure of the tooth. The amount of heat which he uses would only dry out the water in it. He states that at 130° C. the three applications of four and one-half minutes each would bring about certain destruction of all micro-organisms, and that by raising the temperature a hissing sound is produced from root-dryer, say 170°. I should be glad to know how the Doctor determines the temperature of his root-dryer. It has always seemed to me that it would be impossible to do any permanent injury to a tooth from the amount of heat which a patient could tolerate in the way of root-dryer.

Dr. Taft: There is nearly always after a paper is read a lull. Members seem averse to start discussion, possibly because they fear something might be said which will draw criticism upon them. This paper is a good one, it raises some new questions. In reference to water in teeth, I understand the essayist to say that all water in a tooth was in combination with other elements. If this were true, bacteria would not proliferate and grow in dentine. By thorough drying a tooth from the mouth its weight is somewhat reduced, indicating that there was a removal of some material, and we can hardly imagine that a moderate degree of heat would break up this combination of water with other material. The water of crystallization cannot be so readily removed as this, so that as in all other tissues in the body undoubtedly there is a large amount of water which is not in combination with other material. Two reasons for removing water: 1st. Why necessary to remove it? Moisture should not be permitted to remain in cavity because it would render a filling less perfect than it would otherwise be. What degree of heat? Shall there be a thorough drying or should we simply remove water from the surface of the cavity? The latter it seems to me is all that is necessary. Some say, introduce the hot air until the dentine assumes a chalky-white appearance, indicating that the water has been removed. When this occurs it is evident that the desiccation has penetrated somewhat the dentine. This is not necessary, and perhaps would be detrimental to the tooth. The contention in the paper is that this thorough drying or desiccation is likely to render it liable to fracture. I do not believe this, because we have no force to make fracture. Another reason for thorough desiccation is, that if there are bacteria in the mouths of the canaliculi, at that point it would be destructive to them. Another argument for thorough drying is, they cannot proliferate and grow in any structure which is entirely free from water; they must have moisture in which to thrive. This paper also calls forth suggestions in reference to this matter which every one must decide for himself. I regard this paper valuable because it leads to practical points.

Dr. Harvey, of Battle Creek: I would like to ask, what was the maximum temperature of heat applied in these various experiments on teeth?

Dr. Cook: The maximum temperature I really experimented with? To go into detail of all this work would require hours. I carried the temperature in a number of cases to 200° C., but if the temperature rises or is carried to this degree there is a perceptible contraction in the dentinal fiber. There is more of a contraction in the direction towards the periphery from the center. Dr. G. V. Black has stated that the contraction takes place between dentine and enamel, and therefore in giving this I simply brought out some of the points which I had arrived at in the experiments in this line. The temperature, I think, if carried to 170° C. for ten minutes, you would find there would be a contraction and weakening of the tooth. I extracted two bicuspids and kept them at body temperature in saliva. I dried out at 170° C. and applied pressure as Dr. Black has done on his teeth. The one which had been subjected to 170° C., 200 pounds pressure, the one which he had at 325 pounds pressure. This shows that there was a weakening in that particular tooth. At 130° C. I detected no difference in the pressure whatever and could determine no difference in the contraction of dentine, as I stated in my paper. The only thing was that the tubuli were cleared out at 125° C. showing that the spaces in tubuli were larger.

Dr. Kahlo remarked in regard to experimental work out of the mouth, of course we know that it is done under a great disadvantage. They are not as good as I would like to have them. These teeth extracted fresh from the mouth were furnished me by men who extract. I placed them in an incubator at body temperature and under normal conditions. In the drying out of these teeth it seemed that there was no difference in the resistance from those which were extracted from the mouth and put under pressure and those subject to treatment, as paper states. I hope some time to be able to give in detail all the facts of the work and apparatus used. I could get my root-dryer to a certain point, but by raising the heat a little bit we could get the temperature of the point of root-dryer within about 4° or 5° of what thermometer registered. We could not determine the exact temperature by electricity. In regard to the suggestions made by Dr. Taft, I think they were very good. Of course, I did not mean to say we must extract too much moisture. I simply gave the figures as they are registered in the May number of the *Dental Review*, in which I gave several experiments, amount of moisture extracted and the exact account of each experiment. It is only supplementary to this paper.

Dr. Harvey: Mr. President, why I ask this question is the fact that the maximum temperature obtainable in the mouth is not sufficient in my mind to materially affect in the way of fractured tooth structure. Some nine months ago I had occasion to boil some teeth freshly extracted and examine under the microscope to know what effect boiling had upon tooth structure. We found that there was no material change. There was no fracture detected. It is in the elimination of moisture; we find it in nature; it alone brings this change. I do not think it is possible to obtain a degree of 170 in the mouth.

Dr. Cook: Mr. President, I did not mean to advocate that a tooth should not be dried out. I simply mean to say that a tooth is weakened if we raise it to such a degree. If you place a root-canal dryer in a tooth at 170° C., which I did in a number of cases, the patient will feel it very quickly. I believe in the drying out of a tooth; I have done so ever since I have been in practice, but I will not raise the temperature as high as I have done before. I never allow the hissing sound. When you raise the temperature to a degree of ninety, I think you can thoroughly sterilize and desiccate the tooth without injuring it at all. I do not believe it good to go over 130°, that was the impression I wanted to make. I think the most of those who use heat at all use it at a much greater temperature than is necessary. They could get perhaps as good results at a lower temperature, and it will not conflict with the tooth. Regarding the boiling of teeth: we do not use boiling water in the mouth. It is not a very easy thing to do. The way teeth are treated usually with heat is by a dry process. The drying out of a tooth is of importance and I do that, and I make it as dry as possible.

Dr. Clayton: Some one has spoken of water in the dentine. Do they mean in the canaliculi or in the calcific matter? I have been for some time past in the habit of raising the heat of my root-dryer higher than I do now. I cannot see but that I accomplish as much as by using a higher degree of heat in the canal. The water will soon return from the dental tubuli and from cementum. If we raise it high enough to take out all the water and there is no return of water, it seems to me we have weakened very much that root for the placement of crowns. So, for the drying of some, I rely more on bibulous paper. I also destroy the micro-organisms by $H_2 O_2$. If we can destroy the micro-organisms by heat, all right; if we take water away by heat, it will again return. The paper is a valuable one and raises many questions which I think are of interest to us practically.

Dr. F. M. Thompson, of Detroit: The gentleman who has just spoken touches me along a sympathetic line. I have gas and compressed air, and very seldom resort to heat, but do as he says, dry the root and then apply germicide in the canal. In regard to the paper I was a little disappointed, and expected it to take up other subjects. Having investigated the question along this line: The effect of heat on teeth, the subject in general in such cases as over-heat by burr, strip or discs. I lubricate my discs and strips, and do not use over eight burrs a year. What is the effect of heat on tooth substance before filling teeth, if it has an effect on tooth? I do not want you to criticise me. The heating of a tooth with disc or strip is sometimes very great. I lubricate both, but they sometimes get hot. Now it is the heating of a tooth in such a condition as this; does it injure the tooth?

Dr. Cook: It seems to be in the minds of many that the tooth takes up again its normal amount of moisture I do not believe it does. It has been experimentally shown by physiological chemists who have worked on fibrous tissue that they cannot get it to return to its original weight.

One of the peculiarities of the circulation is its not taking up as much water as it had at first. Whether this is true of dentine or not I am not prepared to say. I kept a tooth in an incubator for four weeks, and afterwards removed root-canal filler and weighed tooth, and found it was not any heavier after drying. I kept it in a moist condition for some time. The coloring matter if placed on outside of tooth will not go from periphery to center, but if you place it inside of tooth or pulp-canal it will go from the center toward the periphery.

DR. CLAYTON: One thing called to mind in our experiments on the mouth we find what we have in nature, and that is life which is of so much importance to all of us. Discussion closed.

### "DEGENERATION AND DECAY OF ORAL TISSUES FROM LACK OF EXERCISE."

BY S. B. DEWEY, CLEVELAND, OHIO.

That the oral tissues are degenerating must be apparent to the most casual observer. What is degeneration? The act or state of growing worse, in a decline, as applied to tissues, not up to the standard, not capable of performing their normal functions. Degeneration begins in tissue when repair is less than waste. Decay is gradual failure of health, strength, soundness, in a state of dissolution.

Do we find these conditions in and about the oral cavity and in what tissues? I will consider only such as are exactly observed by the practicing dentist. The lack of full development of the jaws and symmetry in the arrangement of the teeth is due to degeneration or want of normal force to reproduce the structure to its full standard. The first stages of development also retarded owing to the feeble state of the nervous system which controls assimilation and nutrition. The impress upon the embryo will be more or less apparent throughout the developmental period. But if such means as at our command be used to overcome this condition much improvement will be noted. The jaw develops from before backwards, therefore, jaws not exercised by mastication would be less in length than one that had been well exercised. One of the chief factors in the arrest of the development of the jaws is the want of muscular exercise. Blood is not carried to the parts to nourish the bones. The teeth suffer in a like manner; poor-blood supply means want of necessary materials to build up or sustain its enamel and dentine, resulting in early decay. The lack of lateral motion, from little work given the teeth, the alveolar process will be insufficiently developed. The advantages of to-day are not as great as the early races, who lived upon coarse food and which required thorough mastication to prepare it for digestion and assimilation. Large muscles must have correspondingly large osseous systems for their attachment. It will be noticed that those who use their jaws freely have the advantage of a normal development over those unfortunate individuals who study to live without chewing their food. The lower jaw is not alone helped by exercise; chewing must by pressure upon the upper jaw increase the blood supply to the parts, thereby augmenting nutritive changes.

The jaws and teeth were placed in the organism to do a certain kind of work, and on the proper achievement of this function depend the development and future health of these organs. If you wish to prepare man for any particular feat, or to improve any faultily developed part, we put him in training that the organs which are to stand the extra strain may be as fully or perfectly developed as possible. The daily use of a muscle or system of muscles means their perfect development. If we wish sound lungs we must constantly inflate them to their full capacity, not only to-day but every day throughout life. Just as soon as we allow the lungs to do only one-half their part, at that moment they begin to degenerate and must shortly assume a pathological state. May not this apply as fully to the jaws and teeth, *i. e.*, teeth that are not used to grind the food will be improperly developed, weak process, tooth socket poorly nourished, and peridental membrane and gums loose in structure, readily yielding to destructive influences.

As soon as the being passes from the stage of infancy into childhood—infancy ends with the completion of first dentition—

the mouth is equipped with a model that was designed to grind coarse food into a fine pasty mass that may be utilized for the upbuilding and maintenance of the body, and if on entering childhood the child were taught that all food should be thoroughly masticated and incorporated with saliva before passing into the stomach, the advantage gained would be three-fold. As the chewing would give exercise to the jaws and teeth, promoting their growth; secondly, the perfect preparation of food for food digestion would be such as to insure its assimilation; thirdly, under this condition the nutritive changes would be in a balance and structures would be builded that would better withstand unfavorable environment.

It is said that man's ingenuity has weakened his physical condition and shortened his life; this is evident in the organs under consideration. In the preparation of our food his genius is very greatly taxed to supply our tables with food ready to pass at once into the stomach, totally ignoring the mill that nature has provided for grinding, triturating, and intermixing the food with saliva before it shall pass from the outer portal of the digestive tract.

Is it not more to be wondered at that nature's mill should crumble with decay from non-use, the same as the deserted structure reared by man and left by the depleted stream in which vermin run riot, and on which the relentless elements spend their force. The millstone that grinds every day does not become coated with slime and hard incrustations.

The habit of both man and child is to discard all foods that are hard and not ready to be hastily swallowed. The diet should consist of such food as requires chewing and will furnish the proper nourishment to the developing teeth and surroundings, and in later life must serve to clean them and keep the tooth-socket supplied with new blood that its repair may at least equal the waste. In the unused organs the waste is greater than the repair, and as a result we have breaking down of its attachments, pus formation and their loss.

Nature designed that the teeth should be cleansed by chewing solid food, and Dr. Black says: "Keep the margins of the fillings in the interproximal spaces clean by frequent excursions of food over them." Pray tell me how we are going to do this unless our diet is composed of food that requires chewing that these excursions may be made. As dentists, we are appalled at the many children of tender years who require a large number of fillings in their permanent teeth, who, on examination, present teeth and gum margins thickly coated with the soft remains of starchy food in an active state of fermentation. Would this be true if the child lived on food that required chewing?

No matter of what the solid or semi-solid part of our diet consists, let it be thoroughly chewed that it may be intermixed with saliva that its digestive power be not lost or wanting. The saliva being one of the digestive fluids in order to have the process complete and assimilation as nearly perfect as possible, all of nature's aids must be made use of in order that the organs may be in a normal condition. The thorough mastication of the food means to the child clean teeth free from decay, perfectly developed jaws with a mimimum of irregularity, and the building up of the tooth-socket and gum tissues of such quality, phagedenic pericementitis will be a thing unknown in middle and later life. Tough beef and a hard baked loaf would prevent dyspepsia.

How often are we disgusted with our older patients who have applied for relief from so-called pyorrhœa alveolaris, and after we have spent much time in scraping and cleaning to free the teeth and gums of the objectionable accumulation, to have them return after an interval of two or three days with the mouth again reeking with the debris of the past few meals. Often, indeed, do we find this the case, and sorry am I that my listeners are not of this class that they might learn to chew their food and thus clean their teeth, if in no other way. Our patients rely too much on our skill and ability to relieve them of their difficulties, instead of trying to avoid these irregularities they now lead to diseased conditions.

Did you ever see phagedenic pericementism in a tobacco-chewers' mouth? You may find pockets with accumulation therein, but no pus. At least this is my experience, and it is not a little in this line. Some writer on irregularity of the teeth says that irregularity does not exist in the tobacco-chewer's mouth. The habit corrects or prevents it. For some time past I have taken pains to inquire into the habits of those demanding treatment for so-called pyorrhoea, and find it the rule, with no exceptions, that the victim bolts his food which consists largely of mush and sops. Just recently on asking a lady, who is under treatment two or three times a year, as to her food habits. These are her words in reply to the question: "My sister-in-law says she never saw a family so given to sopping their food as ours." And this person in her mouth verified the truth of her statement. The same may be said of some of the lower animals. Contrast the condition of the teeth of the ladies' lap-dog, who is fed on the choice dainties from her table, with the one that gnaws the bones in your back yard; the former has the coated and diseased conditions of the human family, while the latter's teeth are clean, white and firmly set, showing they are amply able to perform the work assigned them by nature. The same is also true of other animals under similar modes of living, viz: The collections in the various zoological gardens.

In the many recent discussions of this vexed topic much has been said about the teeth being the farthest from the centers of nutrition, therefore, suffer in consequence. This being so the more the need of pressing the teeth into active service, requiring a certain amount of work at each meal that new blood may be brought to the parts, thus laying in the supply of nutritive material.

Much stress is also placed on the importance of perfect assimilation of the energized blood that we may have perfect nutrition. Did I hear a voice advocating the more thorough masticating of the food, that it may be easily digested and readily assimilated? We should be teachers as well as healers, ever ready to detect abnormal conditions and vicious habits, and offer the proper advice for their correction. We will find it labor lost trying to reform adult men, but we can place these matters in such light that those having the care of children will feel their importance and enforce their application.

Not long ago as I was talking with a lady, a daughter of one of our ablest dentists; she has two brothers also dental practitioners, all in high standing. This lady was spending some weeks with the family of one of these brothers. There are two sons in this family, aged eight and ten years. These boys, like many of the children of to-day, are under the care of a nurse, and are not fed at the family board. This aunt's visit being a gala occasion the boys appeared at the family table, but mind you, were not served from the same bill of fare. Oat meal mush, bread and butter constituted their dinner. They were about to retire from the dining room before desert was ordered, when the father became furious on finding the crusts of bread on the boys' plates. They had eaten only that which was soft and readily swallowed. The father broke out in this manner: "Is that the way my children are taught to eat? You eat every crust on these plates before you leave this table. Are all children fed this way? Is not this the cause of so much destruction in children's teeth? I am astounded at the conditions met in some of these young mouths, which baffles my skill, and my best efforts seem as naught. We have here found a cause for much of this destruction."

If more of us could see this as our brother did what good work would be inaugurated. Since the above conversation, I was informed by a teacher in an eastern kindergarten that their pupils were required to bring a luncheon of bread and butter and fruit, which is eaten in the presence of the teachers, and they are instructed to see that each child eats the crusts of their bread.

It would seem in this case, at least, that the teachers instead of the dentists had taken the initiative in this important matter. There have been many papers on "Oral Hygiene" in the journals of late, and not one that I have read mentions a thorough mastication of the food as a means of keeping the teeth clean. The eating of solid food helps to clean the tongue and mucous surfaces. The mouths of fever patients do not become free from these coatings until they begin to eat solid food. The coatings on the tongue and mucous membrane contain bacteria, which will excite fermentation in food particles lodging on the teeth. The chewing of coarse food will tear off this film of bacteria that may coat the teeth—will keep them moving so they do not have time to get in their work. Teeth used daily in mastication do not have the accumulations of calculus that are to be seen on the unused organs. Examples are found in mouths where one side is disabled.

Dr. Talbott, says: "If the mouth and teeth were kept absolutely clean, so that chemical changes could not take place, the teeth would certainly never decay, no matter how imperfect the enamel might be developed. If the gums be stimulated in the proper manner and kept in a healthy condition, they are decidedly less liable to become infected with pyorrhoea." He did not suggest a means of obtaining this desirable end. Let me say that proper mastication will keep the mouth clean, nutrition of the teeth and gums more perfect, and that the act will furnish the proper stimulus to keep the gums and tooth-socket in a healthy condition.

The beauty and health of the teeth depend on the perfect harmony of nutrition and absorption, waste and repair. It is better to rely on the chewing to keep the teeth and mucous membrane clean, instead of by the use of drugs. Nature never designed the mouth to be the testing ground for all the antiseptics that the chemist might produce. The constant use of antiseptics lowers the vitality of tissues so as to render them readily assailable by destructive forces.

Atrophy of the jaw from non-use or faulty nutrition is one of the neurotic manifestations—over-development of brain or nervous system, a tendency in modern life. The most rapid growth of the brain occurs during the first seven years, and if we over-stimulate this growth it is at the expense of the osseous system. The most vascular parts are first served with nutritive elements, and if there is not enough to go around the parts farthest from the seat of nutrition (the bones and teeth), the least vascular, must consequently suffer from this extra brain development. Well digested and assimilated food must produce a well nourished nervous system and well developed body in all its parts.

The time that imperfect development of the maxillary bones begin to show and irregularities of the teeth are apparent is between the ages of six and twelve years, at a time when our children are living on a diet consisting largely of cooked ground meals or mush, which robs the jaws and teeth of the exercise which the chewing of more solid food would give them. If habits of exercising the jaws and teeth were acquired at this period, better development would be obtained, and much irregularity would be averted.

It is said that the wholesale extraction of the first molar in the past has caused the arrest of development of the alveolar process as well as of the maxillary bones, for the process and jaws depend for their development largely on the function of the teeth, their articulation and their motion stimulating nutrition and enlarging the arch. The horizontal portion of the lower jaw will be improperly developed because function, one of the most important means of development, is lost, and insufficient room is left for the second and third molars.

If we must needs keep this tooth for work that the jaw may be properly developed, then let us give all the teeth the exercise nature designed them to have, and surely we will have jaws and teeth in greater harmony. As for food that is prepared at the factory and ready to be swallowed at once, why, we will bequeath that to the unfortunates who wear factory teeth that will not decay, and have no peridental membranes to degenerate, therefore cannot suffer for the more thorough mastication of the food. Let me remind you that it is now claimed that the human being can live without a stomach, and should man through misfortune or "degeneracy" be deprived of this supposed important organ, then surely "the mill must grind exceedingly fine," and the provender be well mixed with saliva before it starts on its shortened route.

DISCUSSION.

Discussion opened by Dr. Barrett, of Buffalo: I am a little sorry that the discussion of this paper could not have had more study than I have given it. It was placed in my hands some time ago, but did not read it over until coming here on the train. The author of the paper has called our attention to some important things, and yet I am not unprepared to discuss same. It is not my habit to prepare a paper to be read in a formal kind of a way, and has not been for some time. I must depend on the inspiration from the intelligent faces and snapping eyes before me, not hypnotic (loud laughter). My suggestions must depend on the audience to raise me up to the point from which I will have some illumination from the angels, whether from above or below is of little use to us. There are many important truths in the paper. While theoretically it is indisputable, and while we know very well that exercise must inevitably develop certain tissues, yet there are a good many other factors to be taken into consideration when I study the whole subject. The osseous as it is developed from the very inception of the being himself, the teeth themselves get their conformation; they are from the enamel organ in the first place. The development of the roots will depend more perhaps upon the use made of the teeth, but even these get their contour before the time for the eating of tough meat, which I do not appreciate myself. We increase a vascular supply, and in this way may increase the development of a tooth. I antagonize the paper to do you more good; I do not care for the man who pats you on the back and agrees with everything you say. While it is possible and indisputable that vascular tissue is developed by this use, it is undoubtedly true; but when it comes to the osseous tissue, which is made of inorganic structure, that want of exercise will materially change the contour I do not believe. Yet, he presents another point. He says that the use of the teeth in tissues which shall require exercise of the teeth will develop the structures which surround the teeth and pericemental membrane, and there is wherein the chief beauty of the suggestion which he has given lies. We know the exercise by the chewing of mule meat may influence this. But we must not lose sight of the fact that while exercise will develop the jaw, there is something else which takes place in this—another factor which has an important bearing on the subject—the digestion and nutrition of the part. The first element is to see that the digestion and assimilation is perfect; I do not believe that the mule meat assists in that, so far as the exercise and development of jaws and the teeth go. Comparative anatomy would teach us about that. I am fortunate enough to have more than one skull; "I have only one which influences me in particular, myself." I have a considerable number of skulls of Indians, also Mound Builders, and I have a number of the Sandwich Islanders and Mexican Toltecs. I find that the Indians, largely in the early history of the times, lived almost exclusively by the chase; they lived on meat. The south-western Indians, probably all of them, lived very largely on vegetable food—corn. The ancient Mound Builders, we do not know. The Sandwich Islanders lived on fruit. The Mound Builders have the most perfectly developed inferior maxillary I have ever seen. I can take a lower jaw from the skull of one of them and slip it over mine. The Sandwich Islanders also have large ones. The Sioux have small ones. The Sandwich Islander scarcely ever had pyorrhoea alveolaris. The Indians had a good deal. They exercised their jaws on buffalo meat, their jaws were not extraordinarily developed, but they did have pyorrhoea. We have a gorilla which suffered undoubtedly from pyorrhoea, and yet that fellow, I don't believe, ever laid aside a crust of his bread, or his father demand what they were feeding him on. So the facts of history sometimes belie the most beautiful theories. The exercising of the tooth undoubtedly has a bearing on certain tissues of the tooth, yet the structure of the tooth is brought in before this takes place. It does not materially change the whole thing. Now, there are a good many views that might be taken of this, certain other points might be brought into account. We must remember that function is not one; one thing depends upon another; it may have an independent action, although it derives its nutrition from the same source. All combine together to produce that concert of action which alone can be considered health and not disease. This is true all through life. Go with me down into the dark smithy where the blacksmith works over the ringing anvil and you will see upon his forge the black dust, which is nothing but filth and which covers a kind of smoldering fire. Function takes place in burning it. When he commences to pump the bellows we see the smoke come up and the flame; it breathes and pants and pants until by and by a whole fire springs out. Into the heart and depth of that fire he turns the black iron and finally he takes it out and it is ready to communicate the heat to other bodies. We have function again in the same way, one organ stimulating another. Of course, exercise is very good indeed for the person who needs the stimulus, but for him who is tired and fatigued it is different. The man who has the full set of teeth as nature made them in adult life needs perhaps the stimulus of old beef. But the aged person in whom the function is not as active and who has only a few aged teeth here and there, he is going to eschew instead of chewing these crusts and tough meats. It depends upon the condition you are in; one whose life is passed in the shade and shadow—get him out of doors and into the sunlight and he will develop and grow in his moral and physical nature as well. But to him who follows the plow there is no need to go out and play base ball for exercise or go to the skating rink to develop his legs. To him who has an ordinary civilized life for the development of a tooth which has been in existence all his life, I do not think I should prescribe for him that course of diet. We should endeavor to give to the picture all its beauties and make of it, instead of one continuous chromo, a glorious panorama.

Dr. Copeland, of Ashland, O.: I think I will have to agree with Dr. Dewey in regard to the deposition of lime salts being increased by the increased mastication. Dr. Barrett has acknowledged that it would increase the development of vascular tissue. His theory is that the dentine is developed from the pulp which is a vascular tissue, and lime salts are brought there through the vascular system, and through this development we would get a complete one by that means.

Dr. Barrett: My point was simply this. The gentleman who last spoke does not exactly understand. Take for example, the rebuilding of a church in which the material from old building is used in the new, no new material but just a building up with old material.

Dr. Copeland: At the same time when the crowns of teeth first appear through the gums, the roots are not fully completed. Complete mastication would increase the dentine in that case.

Dr. Stephan, of Cleveland: I think Dr. Barrett has missed the main point which Dr. Dewey intended to bring out. He intended to bring out the point that the vascular tissue was strengthened and brought out. This is one of the reasons for degeneration of these tissues or decrease in vitality, so that they become more easily attacked by diseases, such as pyorrhoea and other diseases to which we are subject. If proper mastication follows, teeth are certainly less liable to be attacked from diseases of that character. If the blood is not thoroughly nutritious, these are the cases where we most frequently or always find pyorrhoea. The blood will be in a low state, or will contain a less amount of nutritious matter, and if this is the case those tissues cannot be restored or kept in a thoroughly healthy condition. I think that was the main point brought out in the paper.

Dr. Douglas, of Rome, Mich.: I think I can see where the structure of tooth can be strengthened by the chewing of crusts. Where exercise is carried on blood is sent, that is the reason why those who have been practicing a number of years and filled with hand pressure have a stronger right hand. It has been inferred that the contour of teeth is complete long before we commence to use them. I am perfectly satisfied that the contour of the tooth in many instances is not complete until several years after the tooth has erupted. I have put in tin fillings in the crown of a number of teeth in the child, say from seven to eight years old, and when he was from seventeen to eighteen or twenty years old, the fillings stood right above the enamel, and on picking it off the place where that filling had laid was a very shallow cavity. What could have held it there I could not imagine, except it was oxidized there. I have kept a record for forty-seven years, and I know that teeth do change their contour, from a filling up as well as breaking down. They become harder from deposits of calcareous salts than the tooth structure, and that must come from the pulp. If teeth are used largely in chewing hard crusts and other hard things, we secure more saliva and have it well mixed, the starchy food is more perfectly digested, and we have a larger supply of nutriment to be carried to the tooth.

Dr. Harvey, of Battle Creek: I take issue with Dr.

Barrett. The mastication of hard foods not only accomplishes the good of cleaning the teeth through this exercise, but prevents dental caries. I can back that up by sub-proof. It should receive the serious consideration of every dentist. The character of food as well as kind is also important. Dr. Barrett says, though the form of the teeth is not changed, yet through the blood supply these teeth are nourished and they can degenerate through a lower vitality, if the character of food which is supplied to the body is of an inferior quality. Mule meat or any other kind of meat will even change tooth structure. I wish to say, also, that another reason for a large amount of caries and diseases of oral cavity, does come from the use of soothing syrups and soft foods. I find that those who live on diet as nature intended do not even need a tooth brush or antiseptics. But it is to us, we, as Americans, who have diseases of teeth; of course, there are inhabitants of China who live on rice, etc., have poor teeth. The skulls of Indians who lived on a vegetable diet and those who lived on a fruit diet, on an island, it was their natural diet, but we must have a different character of food. I believe the exercise in the chewing of material we take into our bodies is the cause of absence of decaying disease of oral cavity. I had prepared a chart showing the effects on the teeth of a cow which was fed on soft foods and slops. It illustrated what exercise would do without the character of food. Hard food did prevent dental caries.

Dr. Barrett: Where did you obtain the facts relating to skulls of Indians?

Dr. Harvey: I obtained it from history. I obtained the fact that Indians did not live on meat but corn.

Dr. Barrett: The finest teeth are those of the Sandwich Islanders. They have less caries than any other aborigines.

Dr. Butler, of Cleveland: I am always interested in hearing Dr. Barrett discuss a question, because he tries to get, as he stated, all the different phases or sides of it; "get on the off-side of the question," "to bring out discussion." Dr. Barrett has a happy faculty of getting on all sides so he can see it all. His criticisms are most helpful. You must remember that Dr. Barrett has gathered these skulls for a specific purpose. At the same time he may have gotten hold of some of the better classes of skulls which do not bring out all the sides of the question. Perhaps this subject has been discussed as much as is due it in justice to others which are to come, yet I think we will all acknowledge that it is one of great importance for the dentist at least, the troubles which we have to combat and for the American people at large, at the least, set us to thinking whether there is anything for us to do to bring about a remedy for this.

DR. KIRK, of Indiana: I have been very much interested in the discussion of the subject. I like the points the essayist makes. What we want to get at in this discussion is, What can we take home to benefit us in our professional life so as to educate our patients in the proper points? I do not agree with all of Dr. Barrett's statements. I like him sometimes and sometimes I do not. I think he is a kind of a contrary mortal sometimes. We may discuss this from the scientific standpoints for our own benefit, but our patients want to know the results of that. We all know that a tooth which is used is better than one not used. A tooth is like an Irishman, it wants something to hit. How many of us in coming in contact with our patients, whether they may be six or sixty, we can tell which side of the mouth is used? Almost invariably you can tell, one side or the other, right or left. The side which is used may be perfectly even and the other side all out of fix. I believe we should obtain all the facts we can, and educate our patients in these points which will teach them to take care of their teeth. On the use and care depends how they will last. I have seen teeth where tough meat would have been the best thing possible for them. I heard it stated in a lecture or talk before the public schools, "We do not need our teeth so much to grind our food as to thoroughly incorporate it with saliva."

DR. SMITH, of Cincinnati: The subject has taken rather a wider range than was designed by the essayist. The use of the teeth for the prevention of caries is another question. I believe with Dr. Barrett that you cannot change the interstitial substance of teeth by use. The investigations of Black that the use of teeth strengthens density of enamel, I doubt very much. We must get away from that idea. The use of the teeth does modify it some, through the theory or facts of evolution the size and number of teeth has been diminished. Coming to the other point of the use of teeth in modifying the vascular tissues, there is no doubt that it does strengthen the attachments of teeth ; you know that in extraction of teeth, those which have been used much are much stronger. I do not want to go into this. A tooth decays because of its environment, and if cleansed by act of mastication, it does not alter the contour or shape of tooth. In reference to caries of tooth, is another question. I did not for one moment suppose that it strengthened the tooth itself, but it is the bones about it which it strengthens.

Dr. Palmer, of Warren, Ohio: The teeth are affected by nutrition. In my early life—and you must allow me to go back a little, because I am of the past—in 1843–44, I spent some time in New York, and New Bedford, which is, as you well know, a port where vessels are fitted out for whaling. Some of these vessels connected with the Sandwich Islands and they brought home specimens from those islands. These Sandwich Islanders afterward came to New Bedford and became citizens there, and dressed just as handsomely in fine clothes and were just as handsomely developed as I have ever seen anywhere, and the specimens of teeth brought there I have never seen equaled in beauty of form and perfection. No artist or nothing in nature could produce the accuracy or beauty of articulation and development of jaws and teeth which I saw that were brought by those people; and now how does it come? The food used by these islanders, before their foods were contaminated with that of Europeans and foreign people; they lived upon fruits and fish, without cooking, ate them raw. They had those beautiful teeth which I have never seen equaled.

Dr. Taft: I do not care to discuss this question to much extent. I have this to say in regard to it. I think it would be well, as a rule, to stick rather more closely to the points contained in the paper than has been done this morning. However, everything which has been said is good, but we lose too much time; it also tends to divert the attention from the points of the paper. The discussion has taken a wide range and brings in many particulars that, though interesting, are not in the paper. One particular, however, referred to by Dr. Barrett, he who opened the discussion, was this, and it has been stated since: That no structural change is brought about in a tooth by the nutrient process. I do not think anybody ever said there was. What do we mean by structural change? Is muscle changed structurally by exercise? Though it may not attain a larger size, there is not structural change in it. A muscle can be enlarged, or it may suffer for want of nutrition, the fiber will be less firm and have less tone and have about same volume. There is a change in the tone of fiber that makes up that muscle. There is no change in the structure of a tooth by nutrient supply, but that there is nutrient supply brought to the dentine I have no doubt, as certainly as to any other portion of the body. That the dentine is nourished, to me has the force of absolute truth. In this discussion reference was made to the supposed statement in the paper which was not a statement—to the development and growth of roots of teeth. If it is to be disputed at all, it is only incidental. It is the support the tooth receives by virtue of its use. Teeth have no circulation or vascular system. There is a pulp in every tooth which is a highly vascular structure, in direct connection with the hard part of the tooth, has not only a structural but possesses a vital connection with it and is a means of nutrient supply to the dentine. If not, why the living tooth or dental canals? There is living substance in these canals and a lining. What are they for? Why did not nature make the tooth perfectly solid? This organic material remains for a definite purpose, and that is for support of the tooth. Where does it get its nutrient supply? The blood flows into the pulp, what for if not to supply nutrition? What is the pulp for? The pulp supplies nutrient material and the dentine takes it up, through and by means of organic material in canals of dentine and this sustains vitality, which adds strength and vigor to the inorganic part. The intertubular substance of the tooth sustains its structure. This is regarded as inorganic material, and this living material permeates that which will receive this nutrient supply. While we have no structural change there is a nutrient change. Some have said that teeth never change. I am surprised to hear such assertions from those who have been long in the profession. Watching the teeth during the period of child rearing, who has not seen the teeth very soft and these same teeth later on become hard and firm? Teeth usually later in life become more dense. During the time of sickness the teeth suffer as do all other organs of the body and do become susceptible to decay more so than before. In reference to mastication, every dentist understands that more or less fully. The tobacco-chewers, for example, have teeth firmly set in the sockets, unless diseased. But the teeth which are properly used in mastication are usually firm. The hardest teeth we have to extract are those well used. This is due, in part at least, to the fact that they have been used as they ought to have been. They have had the exercise. The paper is a very good one and ought to be not only in the hands of the dentist and all patients, and especially those who have the charge of children. It is upon this particular subject that the people are concerned. It begins with the child when he begins to use his teeth; if he contracts a right mastication, the habit will remain with him, and will be correct in the main, during its life, unless it takes up vicious habits by faulty living. This is a very important subject, and the mothers should not only see to it that the child masticates its food but that it uses its teeth right, on both sides, in such a way as to promote the best nutrition. They are all interested in this matter and will put forth their best efforts to secure the best results for the child. This will be a great point gained, will save as many teeth as you ever did by operation, and we will have better teeth for the coming generation, and as we would go on we would have a better generation of people, and the rescue from general disease would be a result which would certainly follow.

Dr. Dewey: Some have made the mistake in stating that it would change the tooth structure. This was not the intention of the paper; the thought was to prevent the degeneration of this tissue after it was formed in growing children; to build up such tissue as in later life would resist the influences which would be brought to bear upon them.

